# How the American Society of Tropical Medicine and Hygiene Can Play a Leadership Role in Climate Action: Results from the 2022 ASTMH Green Task Force Survey

**DOI:** 10.4269/ajtmh.25-0215

**Published:** 2025-11-11

**Authors:** Sapna P. Sadarangani, Laia J. Vazquez Guillamet, Hanna Y. Ehrlich, Bartholomew N. Ondigo, Claire Njeri Wamae, Muhammad Asaduzzaman, Najeeha Talat Iqbal, Theresa A. Townley, Kelly K. Baker, Michele Barry, James Colborn, A. Desiree LaBeaud, Kate Whitfield

**Affiliations:** ^1^ National Centre for Infectious Diseases, Singapore;; ^2^ Department of Infectious Diseases, Tan Tock Seng Hospital, Singapore;; ^3^ Lee Kong Chian School of Medicine, Nanyang Technological University, Singapore;; ^4^ Hospital Medicine, University of Alabama at Birmingham, Birmingham, Alabama;; ^5^ eHealth Group Barcelona Institute of Global Health, Barcelona, Spain;; ^6^ Facultat de Medicina i Ciencies de le Salut Universitat de Barcelona, Barcelona, Spain;; ^7^ One Health Institute, School of Veterinary Medicine, University of California, Davis, California;; ^8^ Department of Biochemistry and Molecular Biology, Egerton University, Nakuru, Kenya;; ^9^ M & D Audubon Consultants, Nairobi, Kenya;; ^10^ Department of Community Medicine and Global Health, Institute of Health and Society, Faculty of Medicine, University of Oslo, Oslo, Norway;; ^11^ Department of Paediatrics and Child Health Biological and Biomedical Sciences, Aga Khan University, Karachi, Pakistan;; ^12^ Division of General Internal Medicine, Creighton University School of Medicine, Omaha, Nebraska;; ^13^ Center for Climate Change and Health Equity, University at Buffalo, Buffalo, New York;; ^14^ Center for Innovation in Global Health, Stanford University School of Medicine, Stanford, California;; ^15^ Clinton Health Access Initiative, Boston, Massachusetts;; ^16^ Department of Pediatrics Division of Infectious Diseases, Stanford University, Stanford, California;; ^17^ Zeroverse, Okinawa, Japan

## Abstract

The American Society of Tropical Medicine and Hygiene (ASTMH) established its Green Task Force (GTF) in 2019 and adopted its Green Statement in 2021 in consultation with the GTF to encourage collective efforts for mitigating climate change as a professional society. The GTF highlighted how climate action aligns with the society’s mission to improve global health in a perspective piece published in 2022. The GTF conducted a survey in 2022 to assess the concerns of the ASTMH community surrounding climate change and the potential role of the society in addressing them. The majority of survey participants reported moderate to extreme concern about climate change as well as a negative impact of climate change on their global health work. Survey results demonstrated strong agreement for ASTMH to lead through interdisciplinary research, capacity building through training and education, development of decarbonization guidelines (particularly for laboratories), and advocacy for wider climate action.

## INTRODUCTION

The American Society of Tropical Medicine and Hygiene (ASTMH) has a diverse global membership, and its key mission is improving global health and reducing the burden of tropical infectious diseases.^1^ Since 2019, the ASTMH Green Task Force (GTF) has identified opportunities to promote sustainable practices within the society and led ASTMH to the adoption of its Green Statement^2^ in 2021. The Green Statement^2^ includes commitment to interdisciplinary research and collaborations that enable environmental sustainability in global health work, raising awareness about climate change and reducing the society’s environmental footprint in line with the international Paris Agreement and the society’s overall mission.^3^ The GTF further developed these ideas in the perspective titled “Why climate action is global health action.”^4^

The ASTMH GTF conducted a survey to inform and support ASTMH’s leadership in developing a climate action implementation plan building on the society’s 2021 Green Statement. The objectives of the survey were to determine the ASTMH community’s 1) attitudes, practices, and perspectives on the impact of climate change on their work in global health; 2) awareness of their professional carbon footprint; 3) views on priority areas for climate action, including gaps and challenges regarding available resources; and lastly, 4) perspectives on ASTMH’s role in response to climate change and climate action.

## MATERIALS AND METHODS

The ASTMH GTF designed the survey in 2022 to fulfill the above-stated objectives after a focused literature review. Green Task Force members piloted a draft survey to ensure readability and comprehension while minimizing the time burden for participants. The final survey link (SurveyMonkey, San Mateo, California) was disseminated using multiple methods: 1) emailed twice to all subscribers of the ASTMH e-list, including members and nonmembers; 2) shared through social media channels from October to December 2022; and 3) promoted in person at the society’s 71st annual meeting in Seattle, WA in November 2022. There were 3,500–3,800 ASTMH members during the survey period from October to December 2022. The demographics of ASTMH members in 2022 were as follows. Approximately 60% were from the Americas (the United States, Canada, and Latin America), 21.1% were from Africa, 7.3% were from Asia, 8.2% were from Europe, and 2.4% were from Australia/Oceania. Approximately 29% were student members (pre- and postdoctoral students) (B. Finn, personal communication).

American Society of Tropical Medicine and Hygiene nonmembers who voluntarily choose to subscribe to the ASTMH have a professional interest in global health and the work of ASTMH, and they may still attend ASTMH annual meetings. Post-ASTMH annual meeting surveys are similarly disseminated to all attendees, both members and nonmembers. We disseminated the GTF survey link to the ASTMH e-list, including members and nonmembers, as these collective responses are informative for the goals of the survey and an opportunity to gather perspectives of interest from the tropical medicine/global health professional sector. The ASTMH is the largest professional organization representing this area. Other surveys may similarly be disseminated to the ASTMH e-list depending on the goals of the survey.

The submission of completed anonymous survey responses to the GTF survey was voluntary. Ethics board review (IRB-300014040) at the University of Alabama at Birmingham deemed the project as nonhuman subjects research.

The survey included demographic questions, multiple-choice questions, and open-ended questions (see the GTF Survey, 2022 in Supplemental Appendix 1). Descriptive statistics for the quantitative data analyses (Microsoft Excel, Microsoft Corp, Redmond, WA) and thematic analyses for the open-ended survey responses (MAXQDA^®^ 2024, VERBI software, Berlin, Germany) were conducted.

## RESULTS

A total of 322 participants completed the survey, a low but comparable response rate with other ASTMH surveys (K. Goraleski, personal communication). More than 70% (*n* = 230/322) of participants were ASTMH members, and 67.5% resided in high-income countries (HICs). The demographic characteristics of survey respondents are shown in Table 1.

**Table 1:** Descriptive characteristics of survey respondents (*N* = 322 unless otherwise stated)

Variable/Characteristic	*N* (%)
ASTMH member (yes)	233 (72.4)
Age, years	
20–29	38 (11.8)
30–39	84 (26.1)
40–49	82 (25.5)
50–59	64 (19.9)
60–79	36 (11.1)
>80	6 (1.9)
Prefer not to answer	12 (3.7)
Race/ethnicity (respondents could select more than one), *n* = 322 answered with a total of 338 responses)	
Black or African American	61 (18.9)
White	168 (52.2)
Asian/Pacific Islander	36 (11.2)
Hispanic/Latin American	30 (9.3)
Indigenous	4 (1.2)
Other	18 (5.6)
Prefer not to answer	21 (6.5)
Gender	
Male	144 (44.7)
Female	170 (52.8)
Nonbinary	2 (0.6)
Prefer not to answer	6 (1.9)
Place of birth	
Africa	67 (20.8)
Asia	36 (11.2)
Australia/Oceania	5 (1.6)
Europe	55 (17.1)
North America	135 (41.9)
South America/Caribbean	24 (7.4)
Professional status	
Employed full time in tropical medicine/global health	214 (66.5)
Medical resident/undergraduate/pre- or postdoctoral student	75 (23.3)
Prefer not to answer	33 (10.2)
Area of residence, *n* = 317 responses	
Low-income country	70 (22.1)
Middle-income country	33 (10.4)
High-income country	214 (67.5)
Place of residence, *n* = 319 participants submitted 323 responses	
Africa	69 (21.6)
Asia	26 (8.2)
Australia/Oceania	8 (2.5)
Europe	36 (11.3)
North America	170 (53.3)
South America/Caribbean	14 (4.4)
Geographic location of collaborators (multiple locations could be selected), *n* = 319	
Africa	211 (66.1)
Asia	119 (37.3)
Australia/Oceania	66 (20.7)
Europe	151 (47.3)
North America	203 (63.6)
South America/Caribbean	111 (34.8)

ASTMH = American Society of Tropical Medicine and Hygiene.

Two thirds of survey respondents (*n* = 182/273) reported noticing that climate change had impacted their work. Most participants (70.8%, *n* = 228/322) provided open-ended survey responses on their experiences (Table 2). The most prominent concerns were the increased incidence and geographic expansion of vector-borne tropical infectious diseases, such as dengue and malaria, and the disproportionate negative and diverse effects of extreme weather events on affected populations and fieldwork arising from local instability and high cost. Additionally, some respondents reported having shifted or amplified their academic focus to include climate change and sustainability, with one of the respondents calling it the “overarching largest global health crisis.” Some participants had consciously changed their work travel habits because of climate change.

**Table 2:** Thematic qualitative analysis of respondents’ perspectives on the impact of climate change on their work in global health

Themes	Main Findings	Examples of Excerpts of Comments from Participants Where Each Excerpt Is from a Specific Participant with the Participant Study Identification Number Mentioned in Parenthesis at the End of the Comment
Changes in disease epidemiology	The most prominent concern among those impacted by climate change was the increased incidence and current or expected geographic expansion of vector-borne tropical infectious diseases, such as dengue and malaria.	“Directly. Cyclones due to the El Niño effect have increased in frequency and directly impacted all of my work … There is no use pretending it does not impact every single disease system we work on.” (participant 57) “The threat of reintroduction of malaria and spread of the disease …” (participant 80) “The unseasonal floods and very harsh summers have led to change in pattern of many diseases in clinical practice … higher snake-human conflict situations in summers and postmonsoon … dengue is a major challenge now which was not the case a few years ago likely linked to climate change.” (participant 304)
Impact on global health work activities and health care services/public health	Participants reported a negative impact of climate change on fieldwork and health care services/public health because of work and travel disruptions secondary to severe and/or unpredictable weather events, decrease of forest areas, and increased local instability and costs as well as impact on personal health. Additionally, some respondents increased workload, and others reported having shifted or amplified their academic focus to include climate change and sustainability.	“The very hot weather makes the working environment unfriendly … A lot of air pollution leads to frequent headaches, poor mood, reduced productivity …” (participant 39) “Moving some of my research toward assessing the impact of climate change and changing weather patterns on emerging IDs.” (participant 54) “Climate change has a negative effect on delivering malaria services in my country … heavier rain causes challenging navigation by road especially unpaved roads … this has a trigger-down effect on the supply of malaria medicines and commodities.” (participant 61) “More extreme weather patterns have delayed implementing activities on the ground and at times have caused us to move activities elsewhere.” (participant 101) “Increased instability (political, environmental, social) in regions that I work in.” (participant 152) “[I]ncreased demand to discuss climate change and climate action.” (participant 182) “More work is needed as arboviruses will become more common in areas that are not currently endemic.” (participant 206) “Weather cancelling flights or shutting down laboratories of collaborators temporarily. People losing samples due to power outages caused by flooding or wind damage to powerlines.” (participant 278) “Less tropical forests for field work.” (participant 302)
Conscious changes in work habits	As a response to climate change, some participants are opting for work arrangements that decrease their commute and long-distance travel, such as working from home, using alternative transport methods (e.g., biking), or being more selective about in-person meetings. In addition, few participants reported looking for alternative, less polluting career options.	“Knowing how much travel contributes to climate change, I have also tried to limit air travel to that which is absolutely necessary. However, having used Zoom meetings for collaborative work through the COVID-19 pandemic, I know the limitations, need to balance and not sacrifice important aspects of global health work, for myself or my collaborators while limiting my carbon footprint.” (participant 1) “Helped me consider the environmental costs of laboratory waste, data processing, and air/car travel.” (participant 9) “Able to use virtual space more and cluster meetings if need travel.” (participant 42) “No longer commuting to work by car; using bus or bicycle.” (participant 113) “Laboratory work has a horrendous climate footprint. Consumables and autoclaving and immense amounts of plastic. Tempting to walk away from this type of work to have a career that is less wasteful.” (participant 272)
Community impact	Participants reported concerns about the disproportionate effect of adverse weather events on target populations, making them victims of economic loss, displacement, and illness.	“I work with refugees and I believe displacement and migration caused by climate events is fast becoming a global challenge we can no longer ignore.” (participant 47) “The seasonal malaria cases have extended beyond the usual months, and these have had tremendous implications on the economy.” (participant 52) “Climate change impacts vulnerable groups more/those who already face disparities (e.g., migrant workers who have to work outdoors for extended periods, children, and pregnant women).” (participant 151) “Its impact is low economic growth in communities due to flooding where the people live on farming.” (participant 256) “The emergence of sporadic high-impact diseases has resulted in the death of many people, catastrophic disease burdens, and significant economic losses that reversed progress gained over decades.” (participant 277)
No impact	Some participants reported no direct impact of climate change on their work in global health. A few expressed skepticism toward the veracity, magnitude, and impact of climate change.	“Inappropriate catastrophizing and distortion (for example stating that climate change drives a larger number and severity of hurricanes despite data directly contradicting this on the U.S. NOAA website) distract from actual threats.” (participant 12) “This is still not clear and needs investigation.” (participant 22) “No impact so far.” (participant 74) “No direct impacts yet—things have not become dire enough yet to where it has forced us to really fundamentally change habits or focus areas for research.” (participant 145) “It has yet to impact my work very directly.” (participant 262)

COVID-19 = coronavirus disease 2019; NOAA = National Oceanic and Atmospheric Administration.

Most respondents (94.1%, *n* = 257/273) expressed concern about climate change, and (93.4%, *n* = 256/274) agreed with the landmark editorial statement on the impact of climate change (Table 3).^5^ The use of climate action resources was limited; only a quarter (25.8%, *n* = 64/248) to a third (33.4%, *n* = 83/248) reported either consulting external experts or using/adapting other guidelines, respectively, whereas others reported not using resources because of lack of knowledge, time, or suitability of resources for their needs (Table 3).

**Table 3:** Respondents’ concern about climate change, awareness of carbon footprint, and perspectives on climate action

Survey Question	*N* (%) as Specified
Opinion/response to the editorial statement,* *n* = 274	
Strongly agree	202 (73.7)
Agree	54 (19.7)
Neither agree or disagree	7 (2.6)
Disagree	5 (1.8)
Strongly disagree	6 (2.2)
Level of agreement with the ASTMH Green Statement adopted in 2021,^†^ *n* = 274	
Strongly agree	194 (70.8)
Agree	59 (21.5)
Neither agree or disagree	8 (3.0)
Disagree	11 (4.0)
Strongly disagree	2 (0.7)
Concern about climate change and awareness of carbon footprint (responses include extremely/moderately), *n* = 273	
How concerned are you about climate change?	257 (94.1)
How knowledgeable do you feel about climate change and associated health impacts?	231 (84.6)
How much does climate change impact your work in global health?	182 (66.7)
How aware are you of your professional carbon footprint?	180 (65.9)
How aware are you of your institution’s carbon footprint?	138 (50.5)
How aware are you of your country’s carbon footprint?	166 (60.8)
Thoughts in response to the following recommendations on climate action made by the Green Task Force (agree/strongly agree responses) in the perspective piece “Why climate action is global health action,” *n* = 271	
Include an ambitious decarbonization plan for ASTMH in the next strategic plan	237 (87.5)
Include members with expertise in sustainability on the ASTMH Board of Directors, including young people from both HIC and LMIC settings to implement the decarbonization goal	233 (86.0)
Provide a platform for ASTMH members to learn carbon literacy and decarbonization from one another	235 (86.7)
Prioritize virtual meetings and when travel is necessary, opt for low-carbon means of transport where feasible	197 (72.7)
Scale up awareness and use of implementation of evidence-based guidance for laboratory personnel and researchers that limits waste and reduces the carbon footprint and laboratory work	250 (92.3)
Respondents’ practice and feedback on resources used, if any, for climate action (participants could select up to 3 responses), *n* = 248	
I do not know of any resources	72 (29.0)
I do not have time to look for resources	39 (15.7)
I use/adapt national or international guidelines	83 (33.5)
I consult with external experts	64 (25.8)
The resources I am aware of are not feasible due to local context nuances	48 (19.4)
The resources I am aware of are not feasible due to the cost required	29 (11.7)
The resources I am aware of are not feasible due to the effort and time required	28 (11.3)
Role that ASTMH should play in climate action and climate change (respondents were able to select more than 1), *n* = 270	
Interdisciplinary research	184 (68.1)
Education	190 (70.4)
Developing guidelines for sustainable practice	162 (60.0)
Advocacy	188 (69.6)
Role model sustainable practice	152 (56.3)
Reduce the negative environmental impact of the ASTMH annual meeting	169 (62.3)
Engaging/enabling ASTMH members/community for innovative solutions	162 (60.0)
No need for ASTMH to act in this area	9 (3.3)
Others	20 (7.4)

ASTMH = American Society of Tropical Medicine and Hygiene; HIC = high-income country; LMIC = low- and middle-income country.

* “The science is unequivocal; a global increase of 1.5°C above the pre-industrial average and the continued loss of biodiversity risk catastrophic harm to health that will be impossible to reverse.”^5^ What are your views on this statement regarding the potential catastrophic effect on global health from global temperature rise and the loss of biodiversity?

^†^ The ASTMH Green Statement is as follows. “Global climate change directly and indirectly impacts the spread of infectious diseases and threatens the public health progress made since the founding of our Society. ASTMH membership recognizes that our activities, both individual and collective, influence climate change and that we can leverage our strengths to be better stewards of our interconnected planet. To successfully carry out our Society’s goals, we commit to (1) Holding ourselves responsible as a community for our impact by reducing the environmental footprint of the Annual Meeting and the *American Journal of Tropical Medicine and Hygiene*, (2) Encouraging interdisciplinary research and partnerships to advance environmental sustainability within our global health efforts, (3) Raising awareness within our Society’s reach.”^2^

The society’s Green Statement (92.3%, *n* = 253/274)^2^ and GTF’s recommendations for ASTMH’s involvement in climate action in the perspective piece^4^ both had strong support. Specifically, 87.5% (*n* = 237/271) agreed with including an ambitious decarbonization plan for ASTMH in the next strategic plan, 86.0% (*n* = 233/271) supported the inclusion of members with sustainability expertise on the ASTMH Board, 86.7% (*n* = 235/271) agreed that ASTMH provides a platform for members to learn about carbon literacy and decarbonization, 72.7% (*n* = 197/271) supported the prioritization of virtual options for ASTMH meetings and low-carbon means of transport when travel is necessary, and 92.3% (*n* = 250/271) supported ASTMH’s involvement in the implementation of evidence-based guidance to reduce our laboratory-associated carbon footprint (Table 3).

Participants strongly supported ASTMH’s involvement in various aspects of climate action, ranging from interdisciplinary research (68.2%, *n* = 184/270) to education (70.4%, *n* = 190/270), developing guidelines for sustainable practices (60%, *n* = 162/270), advocacy (69.6%, *n* = 188/270), role model sustainable practice (56.3%, *n* = 152/270), reduction of the negative environmental impact of the annual ASTMH meeting (62.3%, *n* = 169/270), and the development of innovative solutions by engaging ASTMH membership (60%, *n* = 162/270). Three percent (*n* = 9/270) of respondents expressed that there is no need for ASTMH to act on climate action (Table 3).

Most (70.0%, *n* = 14/20) of the few participants (6.2%, *n* = 20/322) who provided open-ended responses agreed that ASTMH should play an active role in climate action, highlighting the need for sustainable practices at the annual meeting and choosing meeting locations that would save travel-related carbon footprint for most attendees. A few participants still supported in-person annual meetings for the added value that they provide for collaborations compared with online meetings. Conversely, some expressed that other organizations might better address climate action because “it is not really the core business of the ASTMH” and because our actions are unlikely to generate change. A minority of those not supporting ASTMH’s involvement in climate action expressed skepticism about the veracity, magnitude, and impact of climate change (Table 2).

## DISCUSSION

This study examines the views of global health professionals within the ASTMH network on climate change, the impact of climate change on global health work, and the role that ASTMH should play in climate action, considering existing gaps and challenges. Such gaps and challenges include the gap in climate action education, the lack of nuanced guidelines for local context, tool kits for practically implementing scalable climate action solutions in global health research and practice, and other barriers noted by the survey respondents (Table 3). Since this survey, global temperatures have surpassed previous records, making the impacts of climate change starkly evident.^6^

Our survey findings underscore that most participants are concerned about the catastrophic effects of climate change and already are experiencing or expect to experience negative impacts of climate change on their global health work, with a high level of agreement with the society’s Green Statement^2^ and support for ASTMH’s involvement in climate action. These results align with the survey performed by the American Society of Tropical Medicine and Hygiene Committee of Global Health (ACGH) in 2022 that showed that the ACGH membership viewed climate change as a top priority in global health. Despite this, many respondents to the GTF survey report a lack of suitable resources, knowledge, time, and/or empowerment to act.

The ASTMH GTF recommends that the survey results inform ASTMH’s strategic climate plan, emphasizing interdisciplinary research, education, advocacy, developing/disseminating guidelines, and reducing the annual meeting’s environmental footprint. The ASTMH can build and synergize its various professional subcommittees’ activities to enable the climate action activities supported in this survey. For example, the GTF and ACGH have been collaborating to elevate the profile of climate change within the society through joint activities.

Of the possible climate actions recommended in the GTF’s perspective piece, the one with the highest support was the society’s involvement in the implementation of evidence-based guidance to reduce laboratory-associated carbon footprints. A subset of GTF members recently published a review on more sustainable laboratory practices.^7^ The ASTMH could collaborate with laboratory sustainability programs, like My Green Laboratory, LEAF,^8^ the freezer challenge,^9^ or the Green Disc certification,^10^ to provide education and tool kits for our membership. Additionally, ASTMH Board members with climate expertise, including trainee representation from low- and middle-income countries (LMICs) and HICs, could foster inclusive and innovative approaches with high impact.^4^^,^^11^

Although more than 70% of respondents supported the prioritization of virtual meetings or low-carbon means of transport where feasible to minimize the carbon footprint of annual meetings, the need for in-person collaboration was also recognized. Nonetheless, virtual conferences, particularly with newer technologies, can allow far greater participation of trainees and researchers from LMICs and disease-endemic countries.^12^

There are limitations to this study. A self-selection bias for respondents more aware of or sensitized to climate change cannot be ruled out and is inherent to this type of study, particularly given the low response rate (<10% of the total ASTMH network), despite multiple dissemination methods. Additionally, 67.5% of respondents resided in HICs, 57.7% were from the Americas (53.3% from North America), and 52% self-identified as white. Although these results might be expected because ASTMH is headquartered in the United States, 66.5% of respondents were employed full time in tropical medicine/global health with a diverse geographical range of international collaborations, and the geographical distribution of respondents was largely similar to that of ASTMH membership. Our GTF survey had a slightly lower proportion of student/trainee members (23.3%) compared with ASTMH membership, but 10.2% of respondents did not submit a response for the question on their professional status. Although the limited sample size precludes meaningful subgroup statistical analysis to understand the heterogeneity of experiences and perspectives across geographic regions, the qualitative responses in Table 2 provide insights into the quality, range, and diversity of experiences of respondents in different contexts. The survey was designed to inform ASTMH’s strategy and actions pertaining to climate action, building on the ASTMH Green Statement, by bringing forth the perspectives and experiences of members and nonmembers affiliated with ASTMH, the largest professional organization for tropical medicine and global health professionals. It would have limited translation and applicability for global climate policy that is influenced by numerous complex international, whole-of-government, civil society, public, and private sector stakeholders.

## CONCLUSION

In conclusion, the survey shows strong support for ASTMH’s leadership in climate action, carbon literacy education, climate change, and health research for the greater benefit of its unique and diverse global health community working across heterogeneous geographical contexts and challenges. In witnessing the impact of climate change and its exacerbating effect on deepening inequities in vulnerable communities, we argue that it is our professional responsibility to lead solutions.

## Supplemental Materials

10.4269/ajtmh.25-0215 Supplemental Materials

## References

[1] ASTMH, 2024. *American Society of Tropical Medicine and Hygiene Mission Statement*. Available at: https://www.astmh.org/about-astmh#Mission%20Statement. Accessed October 7, 2024.

[2] ASMTH, 2024. *American Society of Tropical Medicine and Hygiene, Green Statement*. Available at: https://www.astmh.org/about-astmh#Green%20Statement. Accessed October 7, 2024.

[3] United Nations, 2024. *The Paris Agreement*. Available at: https://www.un.org/en/climatechange/paris-agreement. Accessed October 7, 2024.

[4] Dumre SP LaBeaud AD Ehrlich H Vazquez Guillamet LJ Ondigo BN Sadarangani SP Wamae CN Whitfield K; American Society of Tropical Medicine and Hygiene (ASTMH) Green Task Force members, 2022. Why climate action is global health action. Am J Trop Med Hyg 107: 500–503. 35895350 10.4269/ajtmh.22-0189 PMC9490666

[5] Atwoli L, , 2021. Call for emergency action to limit global temperature increases, restore biodiversity, and protect health. Lancet 398: 939–941. 34496267 10.1016/S0140-6736(21)01915-2 PMC8428481

[6] Climate Copernicus, 2024. Global-*Average Surface Air Temperature Anomalies Relative* to 1991–2020 for *Each Boreal Summer* (June to August) from 1979 to 2024. Data *S*ource: ERA5. Credit: Copernicus Climate Change Service/ECMWF. Available at: https://climate.copernicus.eu/copernicus-summer-2024-hottest-record-globally-and-europe#:~:text=The%20global%2Daverage%20temperature%20for%20boreal%20summer%20(June%E2%80%93August,2023%20(0.66%C2%B0C). Accessed November 1, 2024.

[7] Bayrau BA Buyukcangaz E Sadarangani SP Ondigo BN Prinzi A LaBeaud AD, 2025. (Un)sustainable science: Greening practices in research, clinical microbiology and veterinary laboratories locally and globally. Open Forum Infect Dis 12: ofae677. 39958524 10.1093/ofid/ofae677 PMC11825989

[8] Bianca R Schell NB, 2024. Lab sustainability programs LEAF and My Green Lab^®^: impact, user experience & suitability. RSC Sustainability 2024: 3383–3396.

[9] My Green Lab, 2024. *International Laboratory Freezer Challenge*. Available at: https://www.freezerchallenge.org/. Accessed November 1, 2024.

[10] Software Sustainability Institute, 2024. *Green DiSC: A Digital Sustainability Certification*. Available at: https://www.software.ac.uk/GreenDiSC. Accessed November 1, 2024.

[11] Asaduzzaman M Ara R Afrin S Meiring JE Saif-Ur-Rahman KM, 2022. Planetary health education and capacity building for healthcare professionals in a global context: Current opportunities, gaps and future directions. Int J Environ Res Public Health 19: 11786. 36142057 10.3390/ijerph191811786 PMC9517386

[12] Whitfield KE Pita AA Jarvis TC Weimen S Bousema T, 2024. Geographic equity and environmental sustainability of conference models: Results of a comparative analysis. Am J Trop Med Hyg 110: 1039–1045. 38574548 10.4269/ajtmh.23-0832 PMC11066347

