# Supplementary material for: How the American Society of Tropical Medicine and Hygiene Can Play a Leadership Role in Climate Action: Results from the 2022 ASTMH Green Task Force Survey
Source: Am J Trop Med Hyg. 2025 Nov 11;114(1):55–61. doi: 10.4269/ajtmh.25-0215 (PMC12781405; doi:10.4269/ajtmh.25-0215)
Supplement: Supplemental Materials [file tpmd250215.SD1.pdf]

Supplementary Data: Supplemental Appendix 1, ASTMH GTF Survey, 2022

Greetings! We, the ASTMH Green Task Force, are keen to learn about your professional experiences regarding climate change and your thoughts on climate action. This is a voluntary, anonymous survey for all aged 21 and older in the ASTMH e-list. Your responses will be kept confidential. Your participation will help inform how the ASTMH Green Task Force and ASTMH as a whole could help address climate change as a community. The ASTMH Green Task Force will share the aggregate findings from the survey. Estimated survey length: 5-10 mins.

Please complete this survey by Day/Month 2022.

Section A: Tell us about yourself

1. Are you a current ASTMH member?
   A) Yes
   B) No

2. Please select your age range:
   A) 20-29 years
   B) 30-39years
   C) 40-49 years
   D) 50-59 years
   E) 60-69 years
   F) 70-79 years
   G) > 80 years
   H) Prefer not to answer

3. Please tell us about your race/ethnicity (may select more than one):
   A) Black or African-American
   B) Asian/Pacific Islander
   C) Indigenous
   D) White
   E) Hispanic or Latino/a
   F) Prefer not to answer
   G) Other (please specify)

4. Please tell us your gender:
   A) Male
   B) Female
   C) Non-binary
   D) Prefer not to answer

5. Where were you born?
   A) Africa
   B) Asia
   C) Australia/Oceania
   D) Europe
   E) North America
   F) South America/Caribbean

6. What is your professional status?
    A) Undergraduate student
    B) Pre-doctoral student
    C) Post-doctoral student/fellow
    D) Medical resident
    E) Employed full-time in tropical medicine/global health
    F) Prefer not to answer

7. I currently live in a:
    a. Low income country
    b. Middle income country
    c. High income country

8. In which country/countries do you live?
    A) Africa
    B) Asia
    C) Australia/Oceania
    D) Europe
    E) North America
    F) South America and the Caribbean

9. In which country/countries do you have collaborators?
    A) Africa
    B) Asia
    C) Australia/Oceania
    D) Europe
    E) North America
    F) South America and the Caribbean

10. What were your air travel habits prior to the COVID-19 pandemic (pre-2020)?

A) I did not travel by air
B) Less than once per year
C) 1-4 times per year
D) 5-11 times per year
E) More than 12 times per year

11. Since 2022, my air travel habits are:
    A) I do not travel by air since the pandemic
    B) Less than once per year
    C) 1-4 times per year
    D) 5-11 times per year
    E) More than 12 times per year

Section B: Climate Change Knowledge and Action

12. In the Editorial titled "Call for emergency action to limit global temperature increases, restore biodiversity, and protect health" published by the *Lancet* in September 2021 co-authored by Editors of many health journals, the authors stated "The science is unequivocal; a global increase of 1.5 C above the pre-industrial average and the continued loss of biodiversity risk catastrophic harm to health that will be impossible to reverse."

What are your views on this statement regarding the potential catastrophic effect on global health from global temperature rise and the loss of biodiversity?

A. Strongly agree, comments:
B. Agree, comments:
C. Neither agree not disagree, comments:
D. Disagree, comments:
E. Strongly disagree, comments:

13. How has climate change impacted your work in global health? Please explain (free text)?

14. The ASTMH Green Statement, adopted in 2021, states:

"Global climate change directly and indirectly impacts the spread of infectious diseases and threatens the public health progress made since the founding of our society. ASTMH membership recognizes that our activities, both individual and collective, influence climate change and that we can leverage our strengths to be better stewards of our interconnected planet.

To successfully carry out our society's goals, we commit to the following:

Holding ourselves responsible as a community for our impact by reducing the environmental footprint of the Annual Meeting and the American Journal of Tropical Medicine and Hygiene

Encouraging interdisciplinary research and partnerships to advance environmental sustainability within our global health efforts

Raising awareness within our society's reach."

What is your response to the ASTMH Green statement:

A) Strongly Agree
B) Agree

C) Neither agree nor disagree
D) Disagree with the following parts of the statement:________
E) Strongly disagree with the following parts of the statement:__________
F) Please explain what you disagree/strongly disagree with:

15. Carbon footprint refers to the total greenhouse gas emissions (GHG), i.e. combination of carbon dioxide and methane, caused directly or indirectly by an individual, organization, event, place, or product as expressed as carbon dioxide equivalent (CO_2e). Check the appropriate box that describes your experience.

	Extremely (A)	Moderately (B)	Somewhat (C)	Not at all (D)	Not applicable (E)
How concerned are you about climate change?					
How knowledgeable do you feel about climate change and any associated health impacts?					
How much does climate change impact your work in global health?					
How aware do you feel of your own carbon footprint through the course of your professional/work activities?					
How aware do you feel of your institution's carbon footprint?					
How aware do you feel of your country's carbon footprint?					

16. Climate actions refers to all activities and policies ranging from individual, institution to the national/global scale that aim to reduce the severity of human-induced climate change and its impacts. (Adapted from UN Sustainable Development Goal 13)

What are your thoughts in response to the following selected recommendations from the ASTMH Green Task Force as shared in the recent perspective piece "Why Climate Action is Global Health Action" (Am J Trop Med Hyg. doi:10.4269/ajtmh.22-0189)?

	Strongly Agree	Agree	Neither Agree nor Disagree	Disagree	Strongly disagree
Include an ambitious decarbonisation					

plan for ASTMH in the next strategic plan.					
Include members with expertise in sustainability on the ASTMH Board of Directors including young people from both HIC and LMIC settings to implement decarbonisation goal.					
Provide a platform for ASTMH members to learn carbon literacy and decarbonisation from one another.					
Prioritize virtual meetings and, when travel is necessary, opt for low-carbon means of transport where feasible.					
Scale up awareness, use, and implementation of evidence-based guidance for laboratory personnel and researchers that limit waste and reduce the carbon footprint of					

laboratory work.					

13. What statement(s) best describe your view on the resources you use, if any, for climate action (Select up to 3)?

    A) I do not know of any resources
    B) I do not have time to look for resources
    C) I use/adapt national or international guidelines e.g. International Organisation for Standardization (ISO) guidelines on Energy Management, Environmental Management, Green House Gas (GHG) protocols, or others (Specify)
    D) I consult with external experts (free text for details)
    E) The resources I am aware of are not feasible due to local context nuances
    F) The resources I am aware of are not feasible due to cost required
    G) The resources I am aware of are not feasible due to effort and time required

14. In your opinion, what role should the ASTMH play in climate change and climate action (may select more than one)?
    A) Inter-disciplinary research
    B) Education
    C) Developing guidelines for sustainable practice
    D) Advocacy
    E) Role-model sustainable practice
    F) Reduce negative environmental impact of the annual ASTMH meeting
    G) Engaging & enabling ASTMH members/community/various sectors for innovative solutions
    H) There is no need for ASTMH to act in this area
    I) Others (specify):_______________

Thank you for your time in completing this survey questionnaire.
